# A study protocol for international consensus guidelines on music therapy for paediatric posterior fossa syndrome

**DOI:** 10.1371/journal.pone.0358571

**Published:** 2026-09-25

**Authors:** James Burns, Jonathan Pool, Karen Twyford, Annette Baron, Melanie Thomas, Anna Menén Sánchez, Eleanor Fallon, Janeen Bower

**Affiliations:** 1 Health Research Institute, University of Limerick, Limerick, Ireland; 2 Children’s Health Ireland, Dublin, Ireland; 3 National Rehabilitation Hospital, Dublin, Ireland; 4 Cambridge Institute for Music Therapy Research, Anglia Ruskin University, Cambridge, United Kingdom; 5 Perth Children’s Hospital, Child and Adolescent Health Service, Perth, Western Australia, Australia; 6 Robert Connor Dawes Foundation, Brighton, Victoria, Australia; 7 Alder Hey Children’s NHS Trust, Liverpool, United Kingdom; 8 Institut Guttmann, Institut Universitari de Neurorehabilitació adscrit a la UAB, Badalona, Barcelona, Spain; 9 Universitat Autònoma de Barcelona, Bellaterra (Cerdanyola del Vallès), Spain; 10 Faculty of Fine Arts and Music, The University of Melbourne, Melbourne, Australia; 11 Music Therapy Department, The Royal Children’s Hospital Melbourne, Melbourne, Australia; East China Normal University, CHINA

## Abstract

Posterior fossa syndrome (PFS) is a common complication following surgical resection of paediatric posterior fossa tumours, characterised by mutism, ataxia, emotional disturbance, and cognitive impairment. Despite significant rehabilitation needs, there is limited evidence regarding effective therapeutic approaches, including the role of music therapy. Music therapy is uniquely positioned to address the multidimensional needs of children with PFS, yet the low incidence and clinical heterogeneity of the syndrome preclude traditional empirical research designs. International consensus guidelines are therefore needed to consolidate clinical knowledge and support consistent, high-quality practice. This protocol describes a two-phase study to develop international consensus guidelines for music therapy practice with children with PFS. Phase 1 involves a pre-Delphi consultation with three regional Advisory Panels (North America, Europe, Australia), each comprising family representatives, music therapists, allied health professionals, and medical practitioners. These panels will identify clinical priorities, family concerns, and interdisciplinary outcomes to inform the development of the survey instrument. Phase 2 uses an international Delphi process with a minimum of 30 experienced paediatric music therapists, proceeding through three to five iterative rounds of item generation, rating, and refinement. Consensus is defined a priori as ≥80% agreement. Quantitative analysis will be conducted in R; qualitative data will be analysed thematically. The study will be conducted and reported in line with DELPHISTAR recommendations. The resulting consensus guidelines will support developmentally responsive, family-centred music therapy assessment and intervention for children with PFS, while identifying priority areas for future research in paediatric neuro-oncology and neurorehabilitation.

## Background

Approximately 60–70% of paediatric brain tumours originate in the posterior fossa [1]. Surgical resection remains the primary treatment modality; however, a well-recognised postoperative complication is posterior fossa syndrome (PFS), also termed cerebellar mutism syndrome (CMS) [1,2]. PFS encompasses a constellation of neurological, cognitive, and behavioural symptoms that emerge following resection of a posterior fossa tumour, predominantly in children. Reported incidence varies widely, from approximately 8% to 50%, depending on definitional criteria, tumour characteristics, and potentially differences in surgical technique and surgeon experience [3–5]. Although the severity, timing of onset, and recovery trajectory of PFS vary substantially, the syndrome has a recognisable core clinical phenotype. PFS is characterised by delayed-onset reduced speech or mutism and emotional lability, commonly accompanied by cerebellar motor signs such as ataxia and hypotonia, as well as cognitive-affect changes. Additional neurological features, including cranial neuropathies and long-tract motor deficits, may occur, with their presence and severity varying according to tumour characteristics, including tumour type, location, and size, as well as surgical factors and developmental stage [6–8].

Children who develop PFS therefore present with complex and multidimensional rehabilitation needs. Although many deficits improve over time, some children experience persistent functional, cognitive, communication, emotional, and psychosocial difficulties that may extend into adulthood [9]. The hallmark feature of PFS is a reduction or complete absence of speech (mutism), typically emerging one to seven days after surgery. This directly impedes the child’s capacity for communication, emotional expression, and participation in their own care [2,10]. Mutism is usually transient, lasting from days to months, and while less common, longer durations of mutism are associated with poorer recovery outcomes [11]. When speech begins to return, it is typically preceded by the recovery of affect and oral intake [12] and followed by a prolonged period of dysarthria. Motor speech deficits are reported to occur in an estimated 98.8% of children with PFS [11,13].

Motor impairments are also common in children with posterior fossa tumours and may be especially prominent in PFS. Abnormal gait and coordination are reported in approximately 60% of children diagnosed with a tumour in the posterior fossa region [14]. Among children who develop PFS, ataxia is particularly prevalent, persisting beyond four weeks post-surgery in more than 88% of affected children and remaining present in approximately 71% at one year after diagnosis [10]. Dysphagia, hypotonia, and cranial nerve palsies further compound the clinical picture [1].

Emotional and behavioural disturbance is a core rather than peripheral feature of PFS. Irritability, emotional lability, and inconsolability are among the most commonly described symptoms [1,15,16], while marked apathy, decreased responsiveness to the environment, physical agitation, and poverty of spontaneous movement may also occur [8]. Crucially, these emotional symptoms do not appear to be only a reaction to the frustration of being unable to speak. The symptoms frequently persist even after speech deficits have improved [15], and children may continue to show emotional and behavioural difficulties years after surgery [17,18]. Longer-term survivors report ongoing challenges with social participation, communication confidence, and emotional regulation [19].

Children with PFS present with a clinical profile that sits at the intersection of paediatric oncology and acquired brain injury, yet does not fully align with typical trajectories in either field: many deficits recover spontaneously, but this recovery progresses alongside active oncology treatment, creating a dynamic clinical picture that the broader neurorehabilitation literature only partially addresses. PFS also occurs within a developmental context in which neurological insult and recovery overlap with ongoing cognitive, motor, emotional, and social development, further complicating rehabilitation planning and outcome measurement [20]. Rehabilitation should therefore be multidisciplinary, longitudinal, and adaptive, responding to the changing needs during treatment and recovery while supporting the child’s ongoing development. However, specific approaches for PFS have not been formally evaluated, leaving limited condition-specific guidance for clinical practice [21].

Music therapy may be particularly relevant for children with PFS because their needs span two traditionally distinct areas of practice. Within paediatric oncology, music therapy often provides psychosocial and supportive care across a prolonged treatment trajectory, with periods of heightened need during acute treatment and hospitalisation [22,23]. In neurorehabilitation, music therapy typically responds to an evolving continuum of care, from critical and acute care through subacute rehabilitation and longer-term recovery, with therapeutic goals adapting as recovery progresses and functional needs change [24]. Children with PFS may require elements of both approaches concurrently, navigating the ongoing demands of cancer treatment while requiring rehabilitation for new and evolving neurological, cognitive, communication, and psychosocial difficulties. Music therapy may therefore offer a means of addressing functional rehabilitation goals alongside the psychosocial needs of children and their families across this evolving trajectory. How music therapy can integrate these approaches and adapt across the course of PFS treatment and recovery has yet to be clearly defined.

Adult models of music processing and emerging paediatric evidence support that music stimulates widespread bilateral neural networks, including regions involved in motor function, language, emotion, and cognition, making it a valuable modality for cognitive, motor, and speech rehabilitation [25,26]. Evidence from paediatric acquired brain injury settings suggests that music therapy can increase arousal and awareness, promote engagement, facilitate neuroplasticity, and support recovery across multiple functional domains [27–29]. However, the low incidence of PFS, the heterogeneity of clinical presentations, and the developmental context of the population make traditional empirical research approaches, including clinical trials, exceptionally difficult to implement, limiting opportunities to build a robust evidence base. To address this gap, the systematic development of international consensus guidelines for music therapy in the care of children with PFS is warranted. Such guidelines have the potential to consolidate current clinical knowledge and expert opinion, identify best practices, and support the delivery of consistent, high-quality, family-centred care across diverse clinical contexts.

## Methods

### Study aim

The aim of this study is to develop international consensus guidelines for music therapy practice with children with PFS. The guidelines will be informed by the priorities and perspectives of families and multidisciplinary professionals and developed through consensus among experienced paediatric music therapists.

### Study design

**Fig 1:**
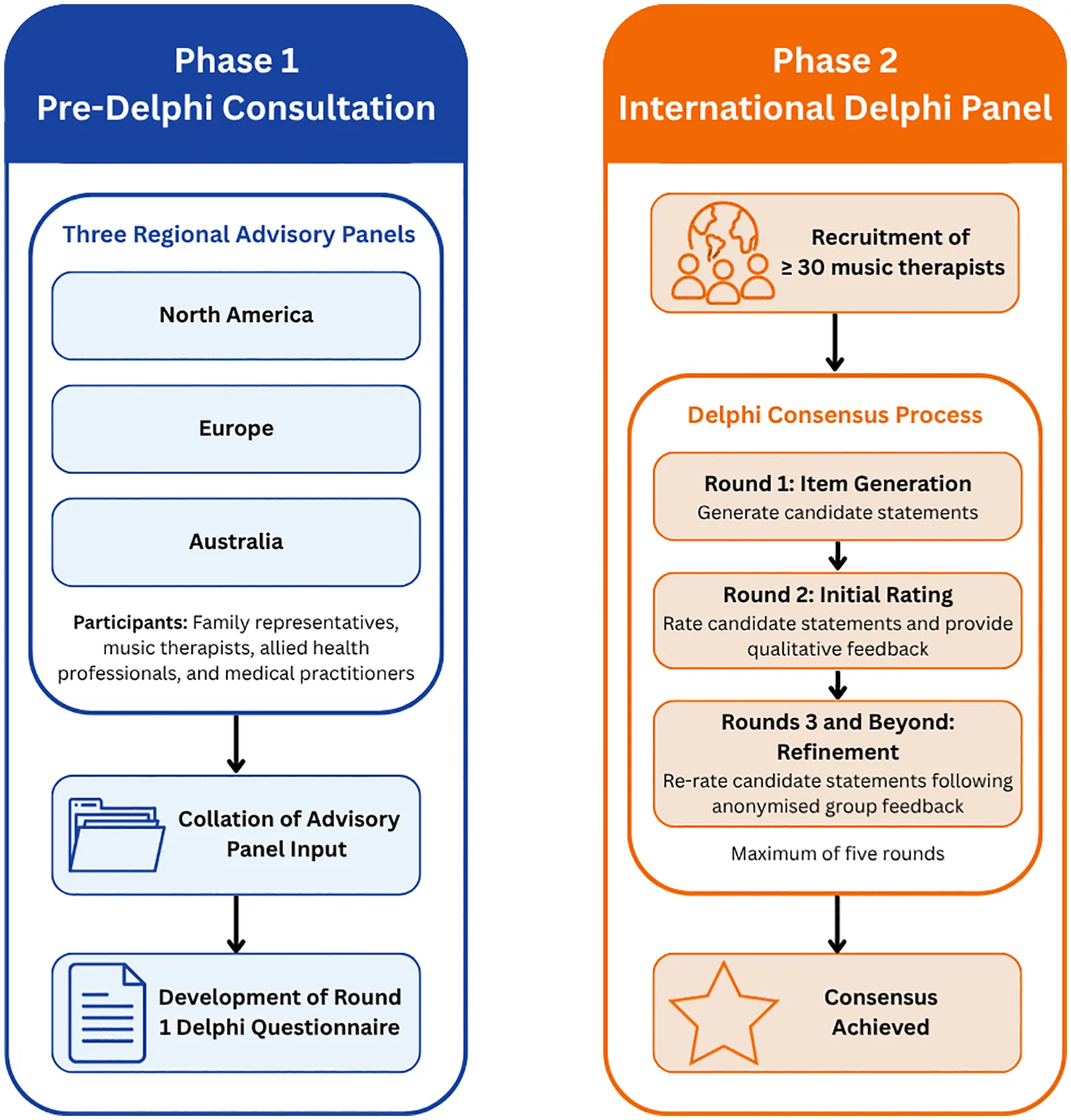
Study design overview.

This study employs a two-phase design. Phase 1 comprises a pre-Delphi consultation with multidisciplinary Advisory Panels, incorporating professional expertise and lived experience to inform the development of the Delphi questionnaire. Phase 2 uses a Delphi process to establish consensus among an international panel of experienced paediatric music therapists on key elements of clinical practice. The study will be conducted and reported in line with the DELPHISTAR recommendations for standardised reporting of Delphi studies in the health sciences [30]. An overview of the study design is presented in Fig 1.

The Delphi method is a structured, iterative process used to elicit expert opinion and establish consensus in areas where evidence is limited [31]. It is widely applied across healthcare and related fields to integrate expert and practice-based knowledge and generate guidelines that support clinical decision-making and implementation [32]. The Delphi process is typically characterised by five features: (i) an anonymous panel of experts; (ii) administration of at least two survey rounds; (iii) use of a standardised questionnaire often incorporating open-ended questions to capture reasoning; (iv) reliance on descriptive statistical analyses; and (v) provision of structured feedback from earlier rounds to allow participants to reconsider or revise their judgements [30,32,33].

A Delphi approach was selected because relevant clinical expertise in music therapy for children with PFS is specialised and geographically dispersed, while the empirical evidence available to guide practice remains limited. Anonymous, iterative participation enables this expertise to be synthesised while reducing the potential influence of dominant individuals on consensus formation. These features are advantageous over consensus approaches that rely on synchronous group interaction or the appraisal of predefined clinical scenarios [34,35].

### Phase 1: Pre-Delphi consultation with advisory panels

Three Advisory Panels will be convened to inform the development of the initial Delphi questionnaire, each comprising participants from one of the following regions: (i) North America, (ii) Europe, and (iii) Australia. This grouping is designed to accommodate scheduling across time zones and facilitate live participation. The selected regions reflect the geographic distribution of the research team’s professional networks and do not represent the full diversity of international practice, which is acknowledged as a limitation. Each panel will include family representatives (parents or caregivers of children who have experienced PFS and received music therapy), alongside music therapists, allied health professionals, and medical practitioners. The panels will identify family priorities, clinical concerns, and interdisciplinary considerations relevant to music therapy practice for children with PFS, thereby grounding the Delphi questionnaire in both lived experience and professional expertise.

### Participants

Each Advisory Panel will consist of approximately 6–8 members (18–24 in total), a size intended to facilitate active participation while enabling representation across relevant stakeholder groups. Participants will be purposively recruited to ensure relevant expertise and lived experience. As Phase 1 is formative, a statistical sample size calculation was not considered appropriate. Consistent with guidance that qualitative data saturation may be achieved with relatively small samples [36], data adequacy will instead be considered iteratively across the three panels, with saturation determined by the extent to which later discussions generate new domains or substantively new perspectives relevant to the development of the Delphi questionnaire. Individuals will be eligible for inclusion if they meet one of the following criteria:

1. Professionals
  - Are aged 18 years or older
  - Hold a tertiary qualification in a relevant health profession (e.g., allied health, nursing, medicine)
  - Have a minimum of five years’ clinical and/or research experience in acute paediatric neuroscience, neuro-oncology, or oncology
  - Have sufficient English proficiency to engage in discussion
2. Family representatives
  - Are aged 18 years or older
  - Are a parent or primary caregiver of a child who has experienced PFS following resection of a posterior fossa tumour
  - The child’s tumour resection must have occurred at least three years prior to recruitment
  - Have sufficient English proficiency to engage in discussion

Due to resourcing constraints, the panels will not be able to accommodate members requiring interpretation or facilitation in languages other than English. This may limit the inclusion of perspectives from non-English-speaking families and professionals.

### Recruitment

Family representatives will be identified through collaboration with community and charitable organisations. Music therapists, allied health professionals, and medical practitioners will be recruited through the professional networks of the study authors and their institutional collaborators.

Potential members will receive an information sheet outlining the purpose of the panel, the scope of their involvement, and the distinction between their advisory role and that of research participants. Interested individuals will be invited to complete an online expression-of-interest form hosted on Qualtrics. Panel membership will be purposively finalised to ensure a breadth of stakeholder, disciplinary, and clinical perspectives, with attention to professional background, clinical setting, geographic location, healthcare systems, service access, and socio-economic contexts. While this approach introduces an element of selection, it is intended to minimise over-representation from any single group. Recruitment through the research team’s professional networks may nevertheless favour individuals with similar professional backgrounds or clinical perspectives, while the restriction to three geographic regions and individuals with sufficient English proficiency may further limit the perspectives represented.

### Advisory panel meetings

Three meetings will be coordinated and facilitated by the second author, one per geographic grouping. All meetings will be conducted virtually via Microsoft Teams and are expected to last approximately 90 minutes. Each will begin with a brief orientation outlining the study aims and the scope of the Delphi process and clarifying the advisory role.

A semi-structured discussion will capture family and professional perspectives on the needs of children with PFS and their families, and priorities relevant to the subsequent Delphi process. Specifically, the discussion will explore: (i) the needs of children with PFS and their families; (ii) the needs that participants consider music therapy may or may not be well suited to address, and the reasons for these views; (iii) appropriate and inappropriate timing for the provision of music therapy; (iv) barriers and challenges to accessing music therapy; and (v) content considered important for inclusion in the guidelines. The facilitator will ensure coverage of core topics while allowing flexibility for members to raise additional concerns or perspectives. Notes will be taken by either the first or final author. A summary will be circulated to members for verification and comment, ensuring that contributions have been accurately represented.

### Development of the initial Delphi questionnaire

Following verification of the Advisory Panel summaries, the research team will review the combined meeting notes and identify recurring discussion points. These will provide an organising framework for the Round 1 open-ended questionnaire. No pre-specified theoretical framework will be imposed; rather, the Advisory Panel discussions will provide an inductive basis for questionnaire development.

The questionnaire will use open-ended questions to elicit free-text responses from panellists regarding their clinical practice and reasoning. At a minimum, the questionnaire will address: (i) music therapy approaches used by participants in their clinical work with children with PFS, including approaches to assessment and intervention and the theoretical frameworks informing these approaches; (ii) participants’ clinical reasoning regarding when music therapy may be initiated, modified, or discontinued; and (iii) their perceptions of potential benefits, challenges, and contextual considerations associated with music therapy for this population.

The draft questionnaire will be reviewed by the research team for clarity, relevance, appropriate wording, and alignment with the study aims. As Round 1 is exploratory and comprises open-ended questions, formal pilot testing will not be undertaken. Any differences regarding question content, wording, or organisation will be resolved through discussion among the research team before the questionnaire is finalised.

### Phase 2: Delphi-based guideline development

An international Delphi Panel of experienced music therapists and researchers will participate in a series of iterative survey rounds to reach consensus on key elements of music therapy practice with children with PFS. In contrast to the Advisory Panels, which incorporate multidisciplinary and lived experience perspectives, the Delphi Panel will consist exclusively of music therapists to enable consensus on discipline-specific practice. Panellists will be paediatric music therapists experienced in neurosciences, oncology, and neuro-oncology. Surveys will be administered via Qualtrics.

At the beginning of each Delphi round, participants will receive an email invitation containing a link to the online survey. Each round will remain open for a defined period to allow response submission, followed by time for analysis and preparation of anonymised group feedback to inform subsequent rounds. Reminder emails will be issued during each round to maximise response rates. Panellists who do not respond within a given round will be considered withdrawn from subsequent rounds. Responses submitted in earlier rounds will be retained in the analysis, as these will have already informed the group feedback provided to remaining panellists. At the conclusion of the Delphi process, all items reaching consensus will be synthesised into a set of international consensus guidelines for music therapy practice with children with PFS.

### Participants

The Delphi method relies on the recruitment of experts, yet there is no universally accepted definition of expertise. In practice, expertise is often inferred from clinical experience, education, or professional recognition, though such approaches introduce potential bias due to non-random sampling [37,38]. For the purposes of this study, expertise will be defined according to the following criteria:

Inclusion criteria:

- Are aged 18 years or older
- Holds a tertiary qualification in music therapy
- Are credentialed or registered music therapist, in line with the requirements of their country of practice
- Have a minimum of five years’ clinical and/or research experience in music therapy within acute paediatric neurosciences, oncology, or neuro-oncology, and direct clinical or research experience with children with PFS
- Have sufficient English proficiency to complete online surveys
- Are able to provide informed consent

Exclusion criteria:

- Prior membership of an Advisory Panel (Phase 1)

### Recruitment

There is no established benchmark for Delphi panel size, with published guidance ranging from 8 to 40 participants [37–40]. Accordingly, this study will aim to recruit a minimum of 30 panellists to participate in the Delphi process.

A call for participation will be disseminated through national music therapy associations and international organisations, including the World Federation of Music Therapy and the European Music Therapy Confederation, supplemented by snowball sampling. These recruitment routes are intended to support broad international participation and capture diversity in clinical approaches and professional perspectives. Interested individuals will complete a Qualtrics registration form capturing key demographic and professional information. Consent will be implied through submission of the form.

Registrations will be reviewed against the inclusion and exclusion criteria, and the first round of the process will commence once the target number of participants has been reached. In the event of oversubscription, panel membership will be purposively finalised to support diversity across geographical regions and the core clinical fields of paediatric neurosciences, oncology, and neuro-oncology. Where multiple registrations meet identical criteria, preference will be given to those with the longest period of relevant professional experience. As with all Delphi studies, panel membership is purposive rather than random, and findings will reflect the views of those who choose to participate, introducing the potential for selection bias [41].

### Data collection and analysis

The Delphi process will proceed through a minimum of three rounds and a maximum of five. Quantitative analysis will be conducted in R (version 4.5.0) and qualitative data will be analysed thematically as outlined by Braun and Clarke [42,43]. At the start of each round from Round 2 onward, panellists will receive anonymised group feedback comprising response distributions and a summary of qualitative comments from the preceding round.

### Round 1: Item generation

In Round 1, panellists will complete the exploratory open-ended questionnaire, providing free-text responses based on their clinical experience and reasoning. Responses will be analysed thematically [42,43] to generate candidate statements for evaluation in subsequent rounds. Coding will be primarily inductive, allowing findings to be grounded in participants’ responses. The first and final authors will independently familiarise themselves with the data and undertake initial coding using NVivo, which will support the organisation of data throughout the analytic process. Coding and interpretations will then be compared and discussed. Differences in interpretation will be resolved through discussion and reference to the source data, with additional members of the research team consulted where required. Related codes and ideas will be grouped to identify broader patterns within the data. These findings will be translated into concise, rateable candidate statements, with overlapping or closely related ideas combined where appropriate.

Credibility and trustworthiness will be supported through the involvement of multiple researchers, maintenance of an audit trail documenting coding and item-development decisions, and transparent documentation of how the qualitative findings inform the resulting Delphi items. The candidate item bank will be reviewed by the wider research team for clarity, relevance, and consistency with the qualitative findings before inclusion in the Round 2 questionnaire. Demographic data will be summarised using descriptive statistics to profile the expert panel.

### Round 2: Initial rating

A structured questionnaire will present the candidate statements generated from Round 1. Participants will rate their level of agreement with each statement using a 5-point Likert scale (1 = strongly disagree; 5 = strongly agree). Panellists will also be invited to provide qualitative comments to justify their ratings or suggest refinements.

### Round 3 and beyond: Refinement

Items not reaching consensus in the preceding round will be re-presented for re-rating alongside the anonymised group feedback. Additional rounds will follow the same procedure until consensus is achieved or a maximum of five rounds has been completed.

### Consensus

While there is no universally accepted definition of consensus in Delphi studies [37,38], most studies operationalise consensus as a specified proportion of participants rating an item within a given range, with thresholds varying from 51% to 100% agreement [37]. A threshold of 80% was selected a priori to provide a relatively stringent criterion for consensus, consistent with recent recommendations for the conduct of Delphi consensus studies [44].

For this study, consensus will be defined a priori as follows:

- Consensus (≥80%): ≥ 80% of respondents rate “agree” or “strongly agree” (4 or 5 on the Likert scale). Statements meeting this threshold will be retained.
- Re-rating (60–79%): Statements will be carried forward for re-rating in the subsequent round.
- No consensus (<60%): Statements will be discarded or, where qualitative feedback indicates that clarification or substantive revision is warranted, revised by the research team for reconsideration in a subsequent round.

In addition to percentage agreement, the median and interquartile range will be calculated and reported for each statement to describe the central tendency and dispersion of responses. These measures will support interpretation of the strength and distribution of agreement, while the predefined percentage agreement threshold will remain the criterion for determining consensus [44].

### Development of the final guidelines

Following completion of the Delphi process, the research team will prepare a draft guidelines document comprising statements that meet the pre-specified consensus criterion of at least 80% agreement. Retained statements will be organised into clinically meaningful domains according to their content. The wording and substantive meaning of each statement will be preserved, with editorial changes limited to improving clarity, consistency, and organisation.

Each retained statement will be presented as a recommendation accompanied by its exact percentage agreement, the number of respondents contributing to the relevant round, and its median and interquartile range. This will allow users to consider the level of agreement associated with each recommendation without introducing post hoc categories of consensus. Statements that do not achieve consensus will not be included as recommendations; where relevant, they will be summarised as areas of uncertainty or priorities for future research.

The draft guidelines document will be circulated to Delphi panellists for final fidelity review. Panellists will be asked to confirm whether the document accurately represents the statements and level of agreement generated through the Delphi process and to identify any inaccuracies or changes in meaning introduced during preparation of the guidelines.

Family representatives who contributed to the Advisory Panels will also be invited to review the draft guidelines for clarity and consistency with the priorities and lived experiences identified during Phase 1. Feedback may inform editorial amendments that do not alter the substantive meaning of agreed recommendations. Any substantive concern that cannot be addressed without changing an agreed recommendation will be documented transparently in the final guidelines.

Formal external validation is beyond the scope of the present study. Subsequent implementation and evaluation studies will be required to assess the acceptability, feasibility, and clinical utility of the guidelines in practice.

### Study status

At the time of writing this manuscript, recruitment for the Advisory Panels is underway, with Advisory Panel meetings anticipated to take place in May 2026. Recruitment for the Delphi Panel is expected to occur between September and October 2026, with Round 1 of the Delphi process anticipated to be completed by late 2026. Subsequent Delphi rounds will take place in early 2027, followed by development and finalisation of the consensus guidelines. Dissemination of the completed guidelines is anticipated by late 2027.

### Ethical considerations

Ethical approval for Phase 1 was granted by the Research Ethics Committee of The Faculty of Arts, Humanities, and Social Sciences at the University of Limerick (2026-01-08-AHSS). Ethical approval for Phase 2 will be sought from the same committee prior to the commencement of the Delphi process.

Participants in both phases may consent to waive anonymity and be acknowledged by name as members of the Advisory or Delphi Panels. However, individual contributions, including specific statements and comments, will not be attributed to named participants.

### Data management

The University of Limerick will act as Data Controller for this study. All data processing will be conducted in accordance with the General Data Protection Regulation (GDPR) and the University’s data governance policies. Data will be collected via the University of Limerick’s institutional Qualtrics platform. Each participant will be assigned a unique identifier code. The linkage key connecting identifiable information to study data will be stored separately in an encrypted file, accessible only to the principal investigators. All datasets used for analysis will be de-identified. Data will be stored on secure, password-protected University of Limerick servers, with access restricted to members of the research team. Data will be retained for seven years following project completion, after which all data will be securely deleted.

### Dissemination plan and data availability

The resulting guidelines will be published in a peer-reviewed journal and presented at relevant national and international conferences. A plain-language summary will be provided to all participants. Guidelines will also be shared with Advisory Panel members, participating organisations, and relevant professional bodies to support translation into practice. De-identified study data and associated research materials will be retained for potential use by future researchers from recognised research institutions. Access may be granted upon reasonable request, subject to ethical review and approval of the proposed use by an independent ethics committee. Data will not be shared where doing so could compromise participant confidentiality.

## Conclusion

Children with PFS present with complex and variable rehabilitation needs, and there is currently limited consensus on the role and delivery of music therapy for this population. This two-phase study will (i) incorporate priorities from families and multidisciplinary clinicians through Advisory Panel consultation and (ii) use an international Delphi process to establish consensus-based guidance on key elements of developmentally responsive, family‑centred music therapy assessment and intervention in this context. The resulting guidelines are intended to support more consistent, transparent, and clinically relevant music therapy practice, while also identifying priority areas for future research and practice development in paediatric neuro‑oncology and neurorehabilitation.
